# The EQ-5D-3L administered by text message compared to the paper version for hard-to-reach populations in a rural South African trauma setting: a measurement equivalence study

**DOI:** 10.1007/s00402-020-03574-5

**Published:** 2020-08-12

**Authors:** Henry G. Burnand, Samuel E. McMahon, Adrian Sayers, Tembisa Tshengu, Norrie Gibson, Ashley W. Blom, Michael R. Whitehouse, Vikki Wylde

**Affiliations:** 1 Avon Orthopaedic Centre, Southmead Hospital, North Bristol NHS Trust, Brunel Building, Bristol, UK; 2 Department of Orthopaedics, East London Hospital Complex, Eastern Cape, East London, Republic of South Africa; 3 Department of Orthopaedics, Royal Victoria Hospital, Grosvenor Road, Belfast, UK; 4 Musculoskeletal Research Unit, Translational Health Sciences, Bristol Medical School, Bristol, UK; 5 National Institute for Health Research Bristol Biomedical Research Centre, University Hospitals Bristol and Weston NHS Foundation Trust and University of Bristol, Bristol, UK

**Keywords:** Hard-to-reach populations, Text messaging, SMS, Patient reported outcome measures, EQ-5D, Measurement equivalence, Rural health services

## Abstract

**Introduction:**

Administering patient-reported outcome measures (PROMs) by text message may improve response rate in hard-to-reach populations. This study explored cultural acceptability of PROMs and compared measurement equivalence of the EQ-5D-3L administered on paper and by text message in a rural South African setting.

**Materials and methods:**

Participants with upper or lower limb orthopaedic pathology were recruited. The EQ-5D was administered first on paper and then by text message after 24 h and 7 days. Differences in mean scores for paper and text message versions of the EQ-5D were evaluated. Test–retest reliability between text message versions was evaluated using Intraclass Correlation Coefficients (ICCs).

**Results:**

147 participants completed a paper EQ-5D. Response rates were 67% at 24 h and 58% at 7 days. There were no differences in means between paper and text message responses for the EQ-5D Index (*p* = 0.95) or EQ-5D VAS (*p* = 0.26). There was acceptable agreement between the paper and 24-h text message EQ-5D Index (0.84; 95% Confidence Interval (CI) 0.78–0.89) and EQ-5D VAS (0.73; 95% CI 0.64–0.82) and acceptable agreement between the 24-h and 7-day text message EQ-Index (0.72; CI 0.62–0.82) and EQ-VAS (0.72; CI 0.62–0.82). Non-responder traits were increasing age, Xhosa as first language and lower educational levels.

**Conclusions:**

Text messaging is equivalent to paper-based measurement of EQ-5D in this setting and is thus a viable tool for responders. Non-responders had similar socioeconomic characteristics and attrition rates to traditional modes of administration. The EQ-5D by text message offers potential clinical and research uses in hard-to-reach populations.

## Introduction

The 2010 Global Burden of Disease study recommended that the development of strategies to quantify the burden of non-fatal health outcomes was a major challenge but also an urgent priority for global health systems [1]. Trauma outcome reporting in Low- and Middle-Income Countries (LMICs) is limited by the inaccuracy and unreliability of hospital statistics as well as low levels of data on patient follow-up [1–3]. Data collection problems are commonplace in South Africa alongside limitations in capacity and resources for research, especially in rural areas [4]. Measuring trauma outcomes is hindered by obstacles to thorough clinical follow-up due to geographical distances, relatively high cost of travel and low motivation amongst the patient population [5]. Even in well-resourced countries with a relatively low incidence of severe trauma, there have been low reporting rates of patient-reported outcomes for trauma [6].

Traditional modes of data collection for patient-reported outcomes are paper based [7]. Substantial technological advances have expanded the options for data collection using devices, such as mobile telephones, and these have overcome some of the physical barriers to data collection [8]. In rural areas of South Africa, even basic outcome data are not well recorded and the rates of clinical follow-up are low [4, 5, 9, 10]. Despite mobile telecommunications advances, the ability to collect data on patient-reported outcomes in remote and ‘hard-to-reach’ settings remains a challenge [11]. Using mobile telephones to collect data represents an interesting opportunity as there has been an increase in widespread usage of mobile phones in South Africa, including rural areas over the past 10 years [12–14]. Recent studies show that healthcare workers and patients are keen to promote their continued use [13–16].

The initial relationships between mobile phone interventions and healthcare in South Africa have been very good. A study of early medical abortion in peri-urban settlements around Cape Town used supportive text messages and a self-administered questionnaire by mobile phone. This was found to be highly acceptable to participants with an acceptability rating of 99% [17–19]. Medication adherence reminders by text message have also been received favourably by patients on anti-retroviral therapy [20, 21]. Other studies have found the use of mobile devices to be particularly suited to survey research in rural areas of South Africa [12]. Set amongst poor existing research infrastructure, the affordability and acceptability of mobile phones in South Africa has made them a viable source for data collection where previously there were scarce or no data.

The EQ-5D is one of the most widely used patient-reported outcome measures (PROMs) [22]. Versions for adult participants include the 3-level response EQ-5D-3L and the 5-level response EQ-5D-5L. It is a generic outcome measure and includes five health dimensions comprising mobility, self-care, usual activities, pain/discomfort and anxiety/depression. It has been applied to and validated for a broad range of health conditions [23, 24]. It has been culturally and linguistically adapted for 172 different language versions (including Xhosa and Afrikaans) which makes it a useful and relevant PROM for remote and rural settings, such as the Eastern Cape Province of South Africa [24].

The traditional mode of administration for the EQ-5D is by pen and paper, but it has also been adapted for other types of self-administration on a screen, such as a tablet computer, via a web-based platform, or personal digital assistant (PDA) format [25]. Furthermore, it can be administered by proxy over the telephone or in a face-to-face interview [26]. The EQ-5D has not previously been available for administration by mobile phone text message/SMS (Short Message Service).

The opportunities offered by mobile technology are not considered to be fully explored [27]. The use of mobile phone text message as a tool for capturing research data is a relatively new concept [28]. The aim of this study was to evaluate equivalence and test–retest reliability of a text message version of the EQ-5D-3L in a rural South African setting.

## Methods

### Study design and setting

This prospective non-randomised cohort study was based in the Orthopaedic Department at Frere Hospital, part of the East London Hospital Complex, South Africa. Data were collected between August 2014 and June 2015.

### Patient and public involvement

Patient and public involvement (PPI) informed the design of this study. Patients and their attendants were asked for their permission to participate in the consultation group at outpatient Orthopaedic clinics. There were two phases for consultations and two groups in each. There were 30 participants in total comprising 13 male and 17 female participants with an age range of 18–68 years.

All consultations were facilitated predominantly in Xhosa by a nurse fluent in both Xhosa and English. The principal investigator was present to make notes. In the first phase, the facilitator used a basic format to facilitate an open discussion about mobile phone ownership and usage, text messaging habits and attitudes to health questionnaires. The cultural acceptability and applicability of a range of questionnaires were explored, including the Quick Disability of Arm, Shoulder, Hand/Wrist (QuickDASH) [29], Patient-Rated Wrist/Hand Evaluation (PRWHE) [30], Patient-Reported Outcomes Measurement Information System-Physical Function/Pain Scores (PROMIS-PF/PS) [31], Short-Form 8 and Short-Form 12 (SF8/SF12) [32] and EQ-5D [26]. The administration of text message questionnaires was then tested with five staff volunteers (three males and two females aged between 25 and 36 years) from the Orthopaedic Department with basic and smartphone handsets.

There was an overall positive reception to health questionnaires in general. It was repeatedly commented that people liked the fact that someone was taking an interest in their injury. There was an equal split between English and Xhosa for language preference when completing a questionnaire and most Xhosa-speaking participants found it easier to read in English but respond in Xhosa. The participants were shown the questionnaires (with translations and assistance) and asked for comments and to indicate a preference. Participants were not very familiar with the concept of rating an activity on a visual analogue scale (VAS) from 1 to 10. The consultations revealed that the number selected on a VAS had been confused with meaning the hour of the day that an activity was performed.

Some of the suggested activities in functional assessment questionnaires considered by the PPI consultations referred to activities not normally performed in a rural South African population. Examples included ‘golf and tennis’ in the QuickDASH and in the PRWHE; the question ‘carrying a 10 lb object in the affected hand’ also caused confusion. Describing the ‘ability’ to perform a task appeared less acceptable than describing the ‘difficulty’ performing a task. The QuickDASH refers to ‘ability’, whilst the PRWHE asks patients to describe the ‘difficulty’ to perform a task, and this caused some confusion [29, 33]. The shorter, more general questionnaires were preferred.

The second phase consultation groups discussed three questionnaires (EQ-5D, PROMIS-PF and SF8/SF12) in greater depth. There are five levels of response statement in the PROMIS-PF and EQ-5D-5L and three levels in the EQ-5D-3L. The PPI participants preferred the three-level EQ-5D-3L questionnaire. A common reason given for this was illustrated by the description of pain in Xhosa culture. Pain is usually described using three levels during clinical consultations: ‘none—*nakancinci*’, ‘a little—*kancinci*’ or ‘a lot—*kakhulu*’. Participants preferred choosing one of three response statements in the EQ-5D-3L and considered it more in keeping with the normal responses given by patients.

The three-level EQ-5D-3L was selected as the preferred questionnaire for testing by administration in text message format. The term ‘SMS’ was preferred to ‘text message’. All participants completed paper versions of the EQ-5D-3L, and then two sample text messages were sent using items from the EQ-5D-3L questionnaire. These were completed satisfactorily by all participants in the second set of PPI consultations. The potential for investigator bias in the PPI consultations was minimised since the majority of discussion was in Xhosa, and therefore, the principal investigator (not a Xhosa-speaker) could not influence it. The positive attitude towards the EQ-5D-3L and the fully completed paper versions were therefore considered independent and unbiased conclusions. The five staff volunteers then received the complete EQ-5D-3L by text message and responded in full.

### Recruitment

Eligible patients included adults with a distal radius fracture, an open tibia fracture or hip joint pathology; owning or with access to a mobile telephone and familiar with the text message function; and able to communicate in Xhosa, English or Afrikaans. Consecutive patients admitted to the hospital Orthopaedic wards were identified by clinical staff and approached for recruitment into this study.

### Text message conversion of the EQ-5D-3L

**Fig. 1:**
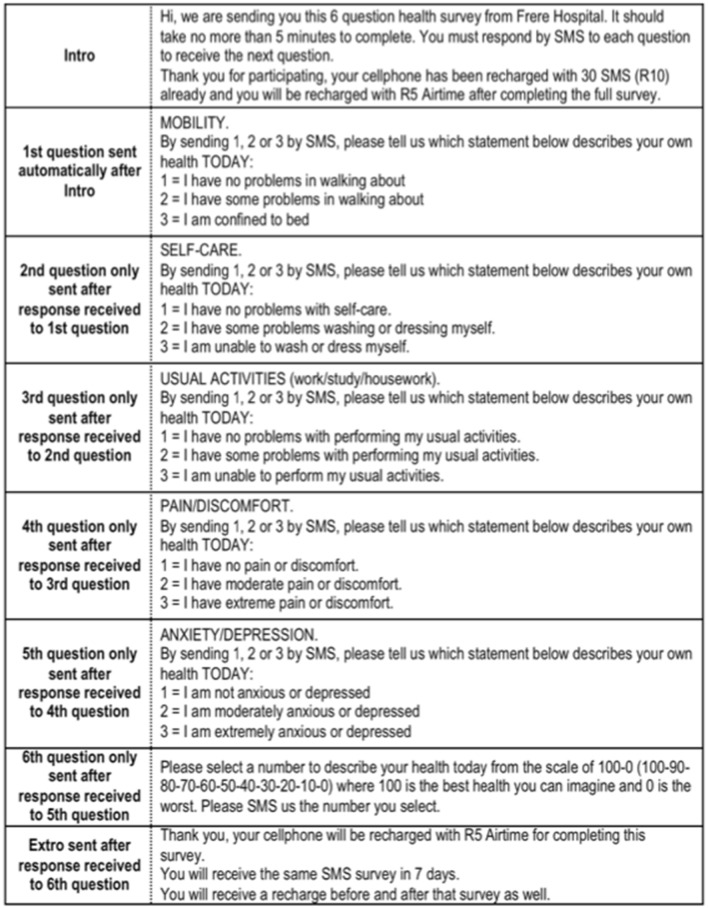
South African English text message version of the EQ-5D-3L (© EuroQol)

Permission was granted by the EuroQol Version Management Committee to test a text message version of the EQ-5D-3L with minimal modification to the original questionnaire. Each question was delivered by text message with additional information contained in the message about how to respond. The questionnaire was preceded by an introductory text message making it clear who was sending the questionnaire, how many questions there were and approximately how it long it should take to complete the full questionnaire (see Fig. 1) in accordance with EuroQol guidance [34].

**Fig. 2:**
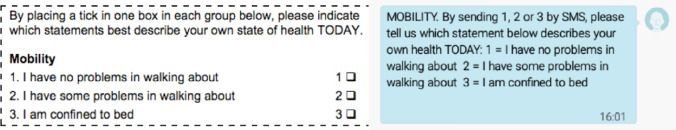
Excerpts from the modified paper and text message EQ-5D-3L (© EuroQol)

In the first section of the EQ-5D-3L paper questionnaire, participants tick a box next to the most appropriate numbered statement for their health status. A number was inserted next to the box (in a similar format to the SF-12 and PROMIS-PF questionnaires) so that participants could use the same numbering system to respond to the text message questionnaire (see Fig. 2) [31, 32]. Patients were still able to tick the box on the paper version and select an equivalent number next to the appropriate statement in the text message version.

The second section of the EQ-5D-3L questionnaire is the EQ-Visual Analogue Scale (EQ-VAS). This graphic scale is specified to be reproduced at a height of 20 cm [34]. Lee et al. changed the EQ-VAS to a verbal numeric scale from 0 to 100 [35]. This adaptation was supported by evidence from equivalence studies showing that visual numeric scales correlated well (*r* = 0.85) with a VAS [36, 37]. In this study participants were invited to select a number on a visual analogue scale from 100 to 0 where 100 is the best health imaginable and 0 is the worst. The full wording of all the EQ-5D-3L items for text message including the VAS is shown in Fig. 1. The original content and wording for each question was maintained as faithfully as possible. This exceeded the maximum number of 160 characters within a single text message. Therefore, two text messages were sent for each question but were received as one text message by the recipient whether using a basic handset or a smartphone.

### Data collection

After giving written informed consent, participants were invited to complete the EQ-5D-3L on three occasions in their preferred language (all approved translations of the EQ-5D-3L). The first iteration was administered in clinic for patients with upper limb fractures or during their inpatient stay after any surgery for lower limb pathology. Participants completed the first questionnaire on paper by themselves. The next two iterations of the EQ-5D-3L were administered remotely as a series of individual text messages 24 h later and again after 7 days. Participants responded to each question of the EQ-5D-3L one at a time by sending a numerical response by text message and were then sent the next question until the series of questions for the questionnaire was completed in full. The responses were entered into an encrypted password-protected database.

A bundle of text messages worth ZAR10 (South African Rand) was credited to participants’ mobile telephones before administering the text message questionnaire to provide them with enough text messages to respond. A compensatory contribution of Airtime worth ZAR5 was credited to the participant’s mobile telephone upon completion of each text message questionnaire. ZAR5 is equivalent to 25% of the proposed hourly national minimum wage for 2019 [38]. Ethics approval was received from the Human Research Committee of Walter Sisulu University Faculty of Health Sciences, Mthatha, Eastern Cape Province, Republic of South Africa (IRB:00007448 HREC:120,009–020 Protocol No: 016/2014) on 2nd June 2014.

### Statistical methods

All statistical analyses were performed using Small Stata 14™ (StataCorp. 2015. Stata Statistical Software: Release 14. College Station, TX: StataCorp LP). A sample size calculation was not performed for this study because there were no baseline data available, and recruitment was constrained by the time constraints and resources available for this study. The dataset was adjusted for the Zimbabwe EQ-5D value set as a proxy since there is no country value set for South Africa. The first statistical analysis was to evaluate the equivalence of the two different modes of administration (paper at baseline versus text message at 24 h). Equivalence was measured by the mean difference of matched pairs (using histograms to demonstrate normal distribution for respondents to both iterations) and the intraclass correlation coefficient (ICC) using a one-way analysis of variance model for agreement between the two different modes of administration. The second analysis was to evaluate the test–retest reliability of the text message administration (text message at 24 h versus text message at 7 days). Test–retest reliability was measured by the ICC of the two text message iterations. Non-responder analysis was evaluated using a Chi-squared test. A *p* value less than 0.05 was considered significant, and acceptable agreement for the ICC was a value greater than 0.70 [39, 40]. Incomplete responses to the questionnaires were considered invalid for analysis as per EuroQol guidance [34].

## Results

### Participants and response rates

**Fig. 3:**
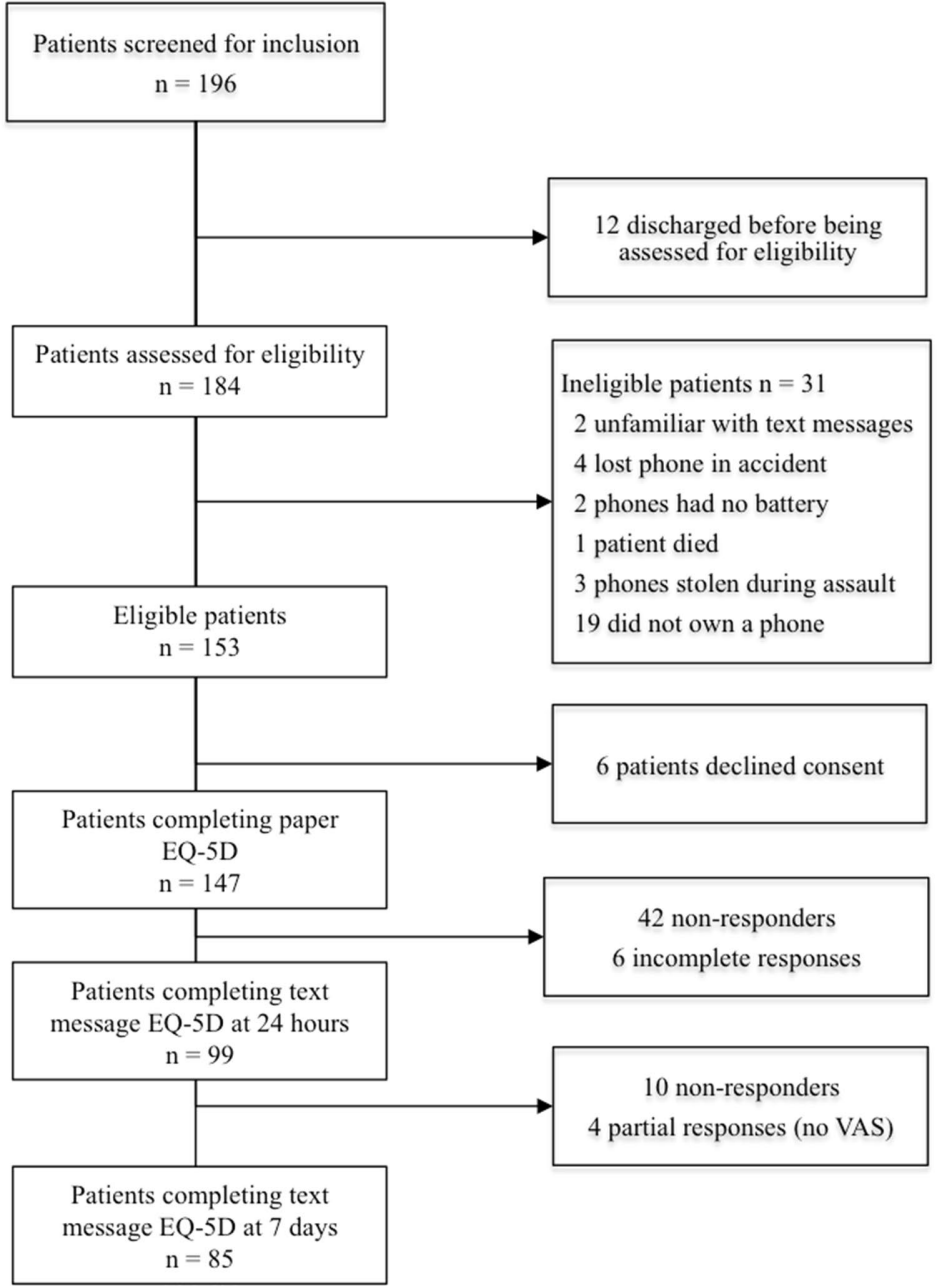
Flowchart showing number of responders at each time interval of the study

There were 184 patients screened for eligibility and 153 were eligible. Reasons for ineligibility included being unwilling to participate, not having a mobile telephone, having lost their mobile telephone or charger and not being familiar with text messages. Of the eligible patients, 147 participants were recruited to the study (Fig. 3). The sociodemographic data and the non-responder breakdown for participants are shown in Table 1. After the initial paper administration of the EQ-5D-3L at baseline, 99 participants (67%) attempted the text message at 24 h (only 95 completed the EQ-VAS as well) and 85 participants (58%) completed the text message at 7 days. All participants (including non-responders and partial responders at 24 h) received text messages at 7 days.

**Table 1:** Sociodemographic and non-responder data for the screened patients

	Non-responder	Responder	Total
Sex			
Female	27	44	71
Column %	56%	44%	48%
Male	21	55	76
Column %	44%	56%	52%
Total	48	99	147
Pearson $\chi^{2}$ = 1.8042, *p* = 0.179			
Age bracket			
18–29 years	6	33	39
Column %	13%	33%	27%
30–49 years	17	35	52
Column %	*35%*	*35%*	*35%*
50 years +	25	31	56
Column %	*52%*	*31%*	*38%*
Total	48	99	147
Pearson $\chi^{2}$ = 8.9492, *p* = 0.011			
Education			
Grade R-9 Education and Training	13	10	23
Column %	27%	10%	16%
Grade 10 + Education and Training	25	71	96
Column %	52%	72%	65%
Not available	10	18	28
Column %	21%	18%	19%
Total	48	99	147
Pearson $\chi^{2}$ = 7.9861, *p* = 0.018			
Employment			
Unemployed	24	46	70
Column %	50%	46%	48%
Not available	2	5	7
Column %	4%	5%	5%
Retired	2	4	6
Column %	4%	4%	4%
Student	0	10	10
Column %	0%	10%	7%
Employed	20	34	54
Column %	42%	34%	37%
Total	48	99	147
Pearson $\chi^{2}$ = 5.4596, *p* = 0.243			
Provenance			
Rural	13	28	41
Column %	72%	58%	62%
Urban	5	20	25
Column %	28%	42%	38%
Total	18	48	66
Pearson $\chi^{2}$ = 1.0732, *p* = 0.300			
Language			
Afrikaans	2	4	6
Column %	4%	4%	4%
English	15	55	70
Column %	31%	56%	48%
Xhosa	31	40	71
Column %	65%	40%	48%
Total	48	99	147
Pearson $\chi^{2}$ = 7.9246, *p* = 0.019			
Pathology	Non-responder	Responder	Total
Distal radius fracture	14	15	29
Column %	29%	15%	19%
Hip joint pathology	15	36	51
Column %	31%	36%	35%
Open tibia fracture	19	48	67
Column %	40%	49%	46%
T otal	48	99	147
Pearson $\chi^{2}$ = 5.09, *p* = 0.08			

The number of responders at each time interval is shown in Fig. 3. Non-responder analyses were performed between the 147 participants who completed the baseline EQ-5D-3L on paper and the 48 patients who did not respond in full to the text message version at 24 h. Non-responders were found to be older (*p* = 0.01), have a lower level of education (*p* = 0.02) and were more likely to speak Xhosa as a first language (*p* = 0.02).

There were 99 matched pairs of the EQ Index on paper and by text message at 24 h. Histograms performed for the matched data demonstrated an approximately normal distribution suitable for *t* testing. There was no statistically significant difference in means of the EQ Index (*p* = 0.95) or the EQ-VAS score (*p* = 0.26) as shown in Table 2. The Pain/Discomfort item demonstrated a significant change between paper and text message iterations.

**Table 2:** Summary statistics of the two different modes of administration (baseline paper versus text message at 24 h)

Item	Baseline paper version mean [95% CI]	Text message at 24 h mean [95% CI]	Paired difference	*p* value
Mobility	1.89 [1.76–2.02]	1.93 [1.81–2.06]	0.04	0.32
Self-care	1.71 [1.59–1.83]	1.70 [1.58–1.82]	0.01	0.81
Usual Activities	2.01 [1.87–2.14]	2.11 [1.98–2.23]	0.01	0.08
Pain/Discomfort	2.19 [2.06–2.31]	2.10 [1.97–2.23]	0.09	**0.04**
Anxiety/Depression	1.62 [1.49–1.75]	1.71 [1.58–1.85]	0.09	0.12
EQ Index	0.54 [0.49–0.59]	0.54 [0.49–0.59]	< 0.01	0.95
EQ-VAS	62.07 [57.20–66.95]	59.94 [55.09–64.78]	2.14	0.26

Bold are statistically significant (*p* < 0.05)

The ICC with 95% confidence intervals (CI) between the EQ Index on paper at baseline and by text message at 24 h was 0.84 (CI 0.78–0.89) which demonstrated acceptable agreement. The ICC for the VAS scores was 0.73 (CI 0.64–0.82) which also showed acceptable agreement.

There were 85 matched pairs who completed the full EQ-5D-3L by text message at 24 h and again at 7 days. There were no significant changes within each item; however, the overall EQ Index and EQ-VAS both demonstrated significant differences as shown in Table 3. The ICC for the EQ Index was 0.72 (CI 0.62–0.82) showing acceptable agreement for the test–retest reliability between 24 h and 7 days. The ICC for the EQ-VAS score was 0.72 (CI 0.62–0.82) also indicative of acceptable agreement for test–retest reliability.

**Table 3:** Summary statistics of text message administration at 24 h and 7 days

Item	Text message at 24 h mean [95% CI]	Text message at 7 days mean [95% CI]	Paired difference	*p* value
Mobility	1.94 [1.81–2.07]	1.82 [1.69–1.95]	0.12	0.06
Self-care	1.72 [1.58–1.85]	1.70 [1.56–1.83]	0.02	0.70
Usual Activities	2.09 [1.95–2.23]	1.98 [1.85–2.11]	0.17	0.06
Pain/Discomfort	2.11 [1.98–2.25]	1.99 [1.85–2.12]	0.13	0.09
Anxiety/Depression	1.67 [1.53–1.82]	1.62 [1.47–1.76]	0.06	0.28
EQ Index	0.55 [0.49–0.59]	0.59 [0.54–0.64]	0.05	**0.01**
EQ-VAS	61.28 [56.59–71.32]	66.69 [61.92–71.46]	5.29	**0.003**

Bold are statistically significant (*p* < 0.05)

## Discussion

### Characteristics of participants and non-responders

This study addressed the challenge of collecting PROM data in a remote rural setting. The sample population was generalisable, but attrition was more common in older participants who spoke Xhosa as a first language and had lower levels of education. This is representative of the association between poorer health outcomes and use of healthcare services in populations with lower health literacy [41]. Text message studies have previously recruited a ‘better resourced’ sample population either through affluent recruiting sites or because they have sampled from a technologically savvy and younger population [7, 42–45]. Any interpretation of results from PROMs should therefore consider the potential for bias towards younger respondents with higher levels of education. One European study evaluating the EQ-5D on smartphones involved posting a mobile telephone to participants and asking them to return the devices after the study [7]. This would have been logistically impractical in the rural South African setting and would have limited the usability testing potential for this mode of administration in hard-to-reach populations [40].

### Equivalence testing

The results of this study have demonstrated acceptable measurement equivalence for the EQ-5D-3L administered on paper and by text message. Our equivalence findings are comparable with other studies evaluating equivalence of the EQ-5D-3L on paper and computer-based administration. Ramachandran et al. investigated measurement equivalence of the EQ-VAS on paper and touch-screen devices and reported acceptable measurement equivalence ICC of 0.75 (95% CI 0.69–0.79) [46]. Lundy and Coons compared paper EQ-5D and Interactive Voice Response (IVR) versions and showed an EQ-Index ICC of 0.89 (lower-bound CI 0.85) and 0.88 (lower-bound 0.83) for the EQ-VAS [47]. In this study, the EQ-Index ICC for paper versus text message was 0.83 (95% CI 0.78–0.89) and the EQ-VAS was 0.73 (95% CI 0.64–0.81). Furthermore, our results are comparable with three meta-analyses investigating measurement equivalence of paper versus electronic modes of administration in which the majority of correlations had an ICC greater than 0.75 [48–50].

### Test–retest reliability

Our results also showed acceptable levels for test–retest reliability of the EQ-5D-3L administered by text message. Shorter time intervals have shown better reliability between electronic questionnaire responses [49]. Lundy et al. excluded responses longer than 72 h from the baseline and reported stronger levels of agreement [47]. The 7-day time interval in this study showed acceptable reliability though this may have been improved by a shorter interval. The change in pain levels in participants around the time of their initial treatment for acute orthopaedic trauma may explain the significant differences between baseline paper and subsequent text message responses in the Pain/Discomfort item.

### Acceptability and feasibility

One of the main challenges facing this study was finding a questionnaire which was acceptable and applicable to the population and feasible for administration by text message without major modifications to the wording and by consequence its psychometric properties. The findings of the first PPI consultations helped select an acceptable and applicable questionnaire using paper versions. The second phase of PPI consultations included testing samples of the questionnaire items by text message. This study benefitted from PPI consultations before and after questionnaire testing. The results from these discussions provided valuable insight into the acceptability and applicability of questionnaires in this setting and administered by text message.

There is currently very scarce data on the longer-term outcomes of patients in a rural South African setting since outcome data are not routinely recorded and clinical follow-up rates are low [4, 5, 9, 10]. In this study, the compensation was ZAR15 per participant per questionnaire, and therefore, the data from this study support a simple and inexpensive mode of data collection for hard-to-reach populations where currently there is no method for PROM data collection.

### Strengths and limitations

Strengths of this study include the use of statistical methodology which follows internationally recognised guidance for measurement equivalence between alternative modes of PROM administration [40]. The PPI consultations helped inform the selection of a questionnaire that would be culturally appropriate and applicable. Liaison with the EuroQol Version Management Committee also enabled the opportunity to test a new version of the EQ-5D-3L in a rural research setting. However, there are limitations that should be acknowledged when interpreting the results. The ICC for measurement equivalence in this study was acceptable but could have been better. Higher precision could have been demonstrated if all the lower boundaries of the ICC confidence intervals had been above the acceptable level of 0.70. This was only shown in the paper versus text message EQ-Index ICC. A meta-analysis of electronic versus paper PROMs found that measurement equivalence was better in studies with time intervals of less than 24 h [49]. The time interval in this study was at least 24 h between paper and the first text message questionnaire, and reducing this may have improved the ICC and lower boundary of the confidence interval. The test–retest reliability in this study may also have demonstrated a higher correlation if a time interval shorter than 7 days had been used [47]. This study was not randomised because it would have been impractical to administer the paper version after the text message version because patients would have been unable to complete the paper questionnaire after leaving hospital. A sample size calculation was not performed prospectively because there were no baseline data available; however, the confidence interval for measurement error in 99 matched pairs is 14% and the paired differences in this study were well within this range [51].

Responding to a text message questionnaire has been considered simpler if the respondent can send a number rather than typing words [43, 52]. This approach was adopted in this study. The questions were modified by adding a number to each statement but keeping the wording, so the conversion of the paper EQ-5D-3L to the text message version was performed with minimal modification according to the framework proposed by Coons et al. [40]. This ‘faithful migration’ followed the interpretation of FDA guidance; therefore, further validation work was not required [40, 48, 53]. A limitation of the text message administration is that participants cannot view all questions at once and cannot review or amend their responses. This has been acknowledged by other studies using the EQ-5D administered on touchscreen smartphones and discussed with the EuroQol Group [7].

The Lancet Commission on Global Surgery 2030 objectives include a proposal for an international consortium on surgical m-Health [54]. This study supports the potential for a mobile telephone-based initiative using text messages for the longitudinal follow-up of patients with orthopaedic pathology. This potential could be expanded to include other surgical disciplines since the EQ-5D is a generic PROM and can also be used as an economic evaluation tool. This would provide a valuable method for benchmarking amongst LMICs and to monitor progress towards achieving global surgery objectives.

## Conclusions

In this study, we have demonstrated acceptable measurement equivalence between paper and text message versions of the EQ-5D and test–retest reliability of the EQ-5D administered by text message. Furthermore, we have found certain cultural issues affecting the acceptability and applicability of PROMs in a rural South African setting. This study also revealed that dropout rates are higher amongst older participants who speak Xhosa as a first language and have lower levels of education, though previous research does not suggest that this is unique to this mode of administration. The results of this study therefore offer potential research uses in data collection for patient-reported outcomes in hard-to-reach populations. Given equivalence is demonstrated, this mode of administration could be used as an adjunct to traditional modes of administration to improve response rates. Evaluation in other socioeconomic settings would enhance the findings of this study.

## Data Availability

The datasets used and/or analysed during the current study are available from the corresponding author on reasonable request.

## Abbreviations

CI: Confidence intervals
ICC: Intraclass correlation coefficient
IVR: Interactive voice response
LMICs: Low- and middle-income countries
PDA: Personal digital assistant
PPI: Patient and public involvement
PROM: Patient-reported outcome measure
PROMIS: Patient-reported outcomes measurement information system
PRWHE: Patient-rated wrist/hand evaluation
QuickDASH: Quick disability of arm, shoulder, hand/wrist
SF8/SF12: Short-Form 8/Short-Form 12
SMS: Short message service
VAS: Visual analogue scale
ZAR: South African Rand
